# The Annual of Scientific Discovery; or, Year Book of Facts in Science and Art—Exhibiting the Most Important Discoveries and Improvements in Mechanics, Useful Arts, Natural Philosophy, Chemistry, Astronomy, Meteorology, Zoology, Botany, Mineralogy, Geology, Geography, Antiquities

**Published:** 1850-05

**Authors:** 


ART. IV.—*The Annual of Scientific Discovery; or, Year Book of Facts in Science und Art*—Exhibiting the most important discoveries and improvements in mechanics, useful arts, natural philosophy, chemistry, astronomy, meteorology, zoology, botany, mineralogy, ge ology, geography, antiquities. Together with a list of recent scientific publications; a classified list of patents; obituaries of eminent scientific men; an index of important papers in scientific journals, reports, etc. Edited by DAVID WELLS, of the Lawrence Scientific School, Cambridge, and GEORGE BLISS, Jr. Boston: Gould, Kendall & Lincoln. 1850.

We had so little space to speak of this work in our April number, that we feel it incumbent on us to renew the subject this month. This is the first number of a work that is destined to be of the greatest utility to scientific men, and we hope that every lover of science, throughout the country, will feel it his duty to encourage the editors to persevere in the good work they have begun.

The editors say: "The idea of preparing the present work was first suggested by the examination of similar works, which have been published in Europe for several years past. We believed that such a work could not fail to be useful to many persons, by enabling them to see at a glance, what has been accomplished during the past year, and thus showing them in what direction they can most profitably apply their labors. The language of Bacon, concerning one branch of science, applies with equal force to all its branches:—'Nothing is of greater efficacy in procuring a stock of new and useful inventions, than to have the experiments of numerous mechanic arts known to a single person or to a few, who might mutually improve each other by conversation; so that by this translation of experiments, arts might mutually warm and light up each other, as it were, by an intermingling of rays.'

"In the preparation of the Annual, nothing has been inserted except upon good authority. While many of the articles have not been previously in print, many others have been furnished directly to us by their authors, but have also been published elsewhere. In the exercise of a proper discretion, we have rejected some articles which it would, perhaps, have been well to retain; but the limits assigned to the work compelled us to omit much that we at first intended to include. We have, however, inserted nearly all that is at once new and important, which is to be found in the standard and scientific publications of America, Great Britain, France, and Germany."

In carrying out these purposes, we think the editors have shown great judiciousness in what they have retained, and we hope they have omitted nothing, connected with the design of the work, of any importance. From a careful examination of the contents, we are impressed with the belief that the work is very complete. We select a few specimens of matters, of interest to medical men.

"*American Microscopes.*—Mr. Spencer, of Western New York, has hitherto been almost the only person in this country who has turned his attention to the manufacture of microscopes, and he has succeeded in producing some instruments of great power and excellence. Most of those, however, at present in use in this country are of foreign construction. About a year since, Mr. J. B. Allen, of Springfield, Mass., having had his attention called to the subject of microscopes, with true Yankee perseverance and ingenuity, set about the construction of one of these instruments. Although he had never seen but one microscope, and that only for a few minutes, and had never seen a piece of glass ground, he devised his own tools and processes, and, in the course of a few months, produced an instrument, which he exhibited to the American Association, at Cambridge, in September. The power of this instrument was about 1,300, and it received the most unqualified commendation of the distinguished men there assembled. Professor Agassiz, after a careful examination of it, made a report, in which he spoke in the highest terms of its excellence. This instrument was purchased by Amos Lawrence, Esq., of Boston, who liberally presented it to the academy at Groton, Mass.

By the advice of Professor Agassiz, Mr. Allen immediately commenced the construction of another microscope, with some improvements suggested by Professor A. This new instrument he completed in about three months. It was submitted to the inspection of Professor Wyman, of Harvard University, who carefully compared it with a similar microscope manufactured by the celebrated Oberhauser, and by him exhibited as one of his best instruments. The American specimen was found to be fully equal, if not superior, to the European, and there can be no doubt that it is the most excellent microscope ever produced in this country.

"It may not be improper to mention, that Mr. Clark, of Boston, has succeeded in producing very fine telescopes, and that excellent chronometers are now manufactured in this country."

"*Further Researches on Electro-Physiology.*—We translate, from the Comptus Rendus of the French Academy, the substance of a paper by M. Matteucci, on electro-physiology. He commences by recapitulating the four principal points from which he started, and which, in some degree, form a summary of his former labors.

" '1. In each cell of the electric organ of fishes, the two electricities become separated under the influence of the nervous activity propagated from the brain towards the extremities of the nerves. A relation exists between the direction and the intensity of the nervous current, and the position and the quantity of the two electricities developed in the cell.

" '2. It has been shown by experiment that the greatest analogy exists between the discharge of electric fishes and muscular contraction. There is no circumstance which modifies one of these phenomena, that does not equally act upon the other.

" '3. The contraction of a muscle develops in a nerve which is in contact with it the cause by which the nerve excites contractions in the nerves throughout which it ramifies. Analogy leads us to consider this phenomenon a proof of an electric discharge developed by muscular contraction, though this has not been decided by experiment.

" '4. The electric current modifies the excitability of the nerve according to its direction: when propagated in the direction of the ramification of the nerve, it destroys its excitability; but when propagated in a contrary direction, it augments it.

" 'I shall now confine myself to communicating a result which I regard as fundamental to the theory of electro-physiological phenomena. By a simple experiment, I have shown that an electric current, which traverses a muscular mass in the direction of its fibres, develops in these filaments a nervous current, which direction varies with that of the electric current, relatively to the ramification of the nerve. This is the reaction of electricity upon the nervous force. In discovering a new and very intimate analogy between the electric discharges of fishes and muscular contraction, I have shown that the nervous current develops the two electricities in a determinate direction, according to its own direction. In a muscular mass, the two electric states, diffused through the elements of its fibres, produce a current, whose direction varying with that of the electric current, is established, like the direction of the discharge in the torpedo fish, by that of the nervous current which excites it. This foundation of the electro-physiological phenomena I have taken great pains to establish.

" 'Whatever may be the nature of the nervous force, it is a fact that this force is propagated in the nerves, sometimes from the brain to the extremities, and sometimes in a contrary direction. It is probable that, when the muscles are contracted by our will, a nervous current is propagated in the direction of the ramification of the nerve; but, on the other hand, the nervous current follows an opposite direction when sensation is experienced by the stimulation of the extremities of the nerve.

" 'I have shown, in my former researches, by experiments, the great difference between the nervous and the muscular substance, as regards the conduction of the electric current. These experiments I cannot repeat, but will confine myself to one, which may be applied to the case in point. This experiment consists in introducing the nerve of a sensitive galvanoscopic frog into the interior of a muscular mass, cut in the direction of its fibres. On passing a tolerably strong electric current through this mass, contractions are never excited in the prepared frog. It is then proved, that, when a muscular mass is traversed by an electric current, the nervous filaments diffused through the mass do not produce any sensible part of this current, so that the effects obtained can be due only to the direct action of the electric current upon the muscular fibre, and to the indirect action or the *influence* of the electric current upon the nervous force.—The following are these effects. If, in a living rabbit, dog, or frog, we expose the muscles of the legs, and pass an electric current from a pile of thirty or forty elements through the muscles, applying one of the poles to the upper and the other to the lower part of the leg,—if the positive pole is placed above and the negative one below, so that the electric current traverses the muscular substance in the direction of the ramification of the nerves, a very powerful contraction is produced, not only in the muscles of the leg, but also in those of the foot.

" 'These results can be explained in but one way. The very powerful contraction excited by the electric current proves the existence of a nervous current passing from the extremities towards the centre, and developed under the influence of an electric current which traverses the muscular mass in the contrary direction to that of the ramification of the nerve. These conclusions have an important connection with the law of electric discharges in fishes, which arise from the production of a nervous current by the stimulation of the nerve, which is distributed in the organ. But in the experiments described, a nervous current is produced by the electric discharge passing through the muscle. In the *discharge of the torpedo, therefore, the electric states are produced by the animal, while in the experiment the nervous current is produced by the influence of the electric current.*' "

"*Physiological Acoustics.*—In order to ascertain the causes of the same body communicating to our ear different tones at the same time, M. Duhamel has made the following experiment. A caoutchouc thread connected consecutively with different points of an oscillating plate, producing simultaneously two or three notes, was conveyed to one ear, while the other was stopped. He convinced himself that an impression of sound could arrive at the ear in this manner only, and yet all the notes were audible at the same time at all the different points. Hence Duhamel concludes, that if the oscillatory motion of one point be decomposed into several others, the ear is affected in the same manner, whether the component movements emanate simultaneously from several neighboring points, or from one point only."

"*Annihilation of the Smell of Musk by the Ergot of Rye.*—The emulsion of bitter almonds possesess the property of annihilating the smell of musk, and most of the cyanic preparations evince the same power. According to M. Mertot, a druggist of Bayeux, in Normandy, ergot of rye will produce the same effect. 'I had, said he, 'to prepare a number of pills, containing both musk and ergot,—hardly were the two substances mixed, than the smell completely went off, so much so, that the patient, who was not aware of the nature of the pills, only noticed the musk by the effects of flatulency."—*Journal de Chimie Médicale.*

These extracts will serve as something of an indication of the character of this excellent and very useful book. We hope that no one of our readers will hesitate about ordering the work. The remittance of one dollar to the publishers, Gould, Kendall & Lincoln, of Boston, will secure the transmission of the book, by mail, free of postage.

